# Proximie in the operating theatre: evaluation of a virtual operating platform for medical student education

**DOI:** 10.1308/rcsann.2024.0028

**Published:** 2024-05-24

**Authors:** DC Schramm, A Abdul-Hamid, J Ramsden, R Mathew

**Affiliations:** ^1^ Medical Sciences Division, University of Oxford, UK; ^2^ Oxford University Hospitals NHS Foundation Trust, UK

**Keywords:** Surgical procedures, Operative, Medical education, Ear, nose and throat diseases, Educational technology, Students, medical, Distance learning

## Abstract

**Introduction:**

Medical students often hesitate to enter the operating theatre because of poor visibility of the surgical field and anxiety about the theatre environment. In addition, ear, nose and throat (ENT) surgery is underrepresented in many medical curricula. Virtual systems like Proximie offer flexible viewing of surgeries with surgeon commentary, potentially addressing these issues.

**Methods:**

This descriptive survey study aimed to evaluate the use of Proximie as a surgical education tool for delivering ENT teaching to medical students. Live ENT procedures were recorded at the ENT Department of the John Radcliffe Hospital and shared with interested clinical medical students through Proximie accounts. Students were added to a private group chat to ask questions and provided feedback through structured forms, assessing procedural effectiveness and the platform's technology. Live-streaming and recording of procedures were facilitated by ENT surgeons providing commentary.

**Results:**

Conducted over four virtual theatre days, the study gathered 52 responses: 96% of students rated Proximie's educational value as 4 of 5 or higher; 57% preferred the virtual experience over physical attendance because of its convenience and the improved view of the surgical field. Students valued the live commentary and showed interest in using Proximie for a broader range of surgeries. Suggested improvements included fixing technical issues, better communication of theatre lists, and expanding surgical specialty coverage.

**Conclusions:**

Proximie has been highly rated by medical students for its effective and engaging approach in the instruction of surgical skills, underscoring its value as an educational tool. Future research is needed to formally assess knowledge acquisition and retention across multiple surgical subspecialties. This work is the first step towards evaluating the utility of virtual operating theatre platforms for medical student education.

## Introduction

Medical students can feel apprehensive about entering the operating theatre when on surgical placements. This reduces their surgical exposure during medical school. Commonly cited reasons for this apprehension include intimidation, a lack of visualisation of the surgical field and a lack of clear learning objectives.^1–4^ Other reasons have centred around variable experiences with staff and inadequate preparation or background.^5^ Medical students often have limited exposure to ear, nose and throat (ENT) surgery within their curriculum.^6–8^ Virtual recordings of the operating theatre have the potential to address both issues. Virtual operating theatre sessions have been shown to improve student knowledge and self-reported confidence.^9,10^ Surgeons that are interested in teaching are more likely to host these virtual operating sessions, which can improve the learning environment for medical students.^11^ ENT is particularly advantageous for virtual operations, because microscope and endoscope feeds can be streamed to students. Virtual streaming of ENT procedures has been tested during the COVID-19 pandemic with some success.^12^ Because operating theatre space limits the number of students able to attend a given surgery, a virtual theatre environment allows more students to access ENT surgeries remotely while still allowing interaction with the operating team.

Proximie is positioned to offer educational live-streaming of procedures by providing filming equipment on a secure platform and allowing streaming of multiple video feeds in high resolution. As a purpose-built operating theatre platform, a Proximie subscription includes the purchase of filming and audio equipment, and use of their online software. Students or surgical trainees can be added to individual groups, where they will be notified about virtual sessions. Operations with multiple video streams can be saved so that those who are unable to attend live sessions can access the recording at a later stage.

## Purpose

The purpose of this pilot study is to explore the use of Proximie in delivering ENT surgical teaching. Our primary research question was whether the Proximie platform is a useful tool for medical students to join live operating theatre lists remotely. Our secondary question was whether the content delivered through remote surgical operations is effective at teaching ENT surgical procedures.

## Methods

Procedures were chosen that focused on key topics for medical students and were typically under 1h in length. Our initial aim was to include tonsillectomies, myringoplasties, grommets, adenoidectomies, endoscopic septoplasty, manipulation under anaesthetic of nasal bones, functional endoscopic sinus surgery, microlaryngoscopy and bronchoscopy, and panendoscopy.

The date and details of the procedures were sent via email to the entire University of Oxford clinical medical student body (Years 4–6 of the undergraduate programme and Years 2–4 of the graduate-entry programme). The entire student body was notified via email on multiple occasions. Each student that expressed interest was given access to a WhatsApp group chat and a Proximie account to watch the operating sessions. Student status was verified by signing up with a valid University of Oxford email address. The WhatsApp group chat was used to remind the students of operating theatre days 1–2 days before they occurred. Moreover, this WhatsApp group was used to inform students of the timings of operations on the theatre day.

Patients and/or their guardians (where applicable) were consented for these procedures being live streamed over Proximie and saved for medical student viewing. Consultants or surgical trainees consented patients on the day of surgery and recorded this on the consent form under the section pertaining to medical students. Recording was delivered through a combination of two free-standing cameras and an endoscopic or microscope feed (Appendix 1 – available online). During the live procedures, the commentating surgeon wore an earpiece which was connected to the hosting Proximie computer. The commentating surgeon responded to questions and provided commentary throughout the operation. Typically, the commentating surgeon was assisting during the procedure; however, the primary surgeon hosted some sessions when they were comfortable doing so.

At the end of each session, students were asked to complete a structured feedback form that was sent by email. Students who had completed a form previously were asked to fill in a separate form for each session they attended. These questions aimed to address our two research questions and are listed in Appendix 2 (available online). This feedback form collected student demographics, information about the procedure and information about the session's effectiveness using either a differential scale with bipolar descriptors (e.g. 1=poor 5=excellent) or free text. The questions on surgical learning focused on students' personal perspectives while observing surgical procedures with commentary, rather than objectively assessing knowledge acquisition or retention. The aim was to obtain feedback from 50 medical students. There were no introductory videos shown, despite this question being asked on the feedback form.

## Results

### Procedures and attendance

We ran four operating theatre days between March and September 2023, gaining a total of 52 responses. A planned session had to be cancelled on the day because of issues with the Proximie video capture cards. Seventeen ENT procedures were filmed. The procedures that were shown to students included two adenotonsillectomies, an adenoidectomy, an atticotomy, a cochlear implant electrode replacement, four microlaryngoscopies, two nasal endoscopies, a rigid bronchoscopy with foreign body retrieval, a myringotomy with grommet insertion, a tonsillectomy and three tympanoplasties (including mastoid surgery).

**Figure 1:**
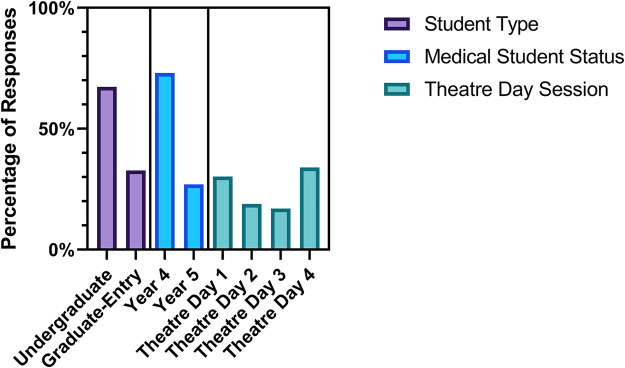
Percentage of survey responses given for medical student type (undergraduate or graduate-entry), student year and theatre day attended. Year 4 from undergraduate entry and Year 2 from graduate-entry students are displayed together as ‘Year 4’. Year 5 from undergraduate entry and Year 3 from graduate-entry are displayed together as ‘Year 5’.

The students that attended were mainly on the undergraduate course and in their first clinical year (Year 4). The responses to the survey were highest on the first theatre day and the most recent theatre day in September, with the other two theatre days having reduced numbers of participants (Figure 1).

### Educational value of Proximie

**Figure 2:**
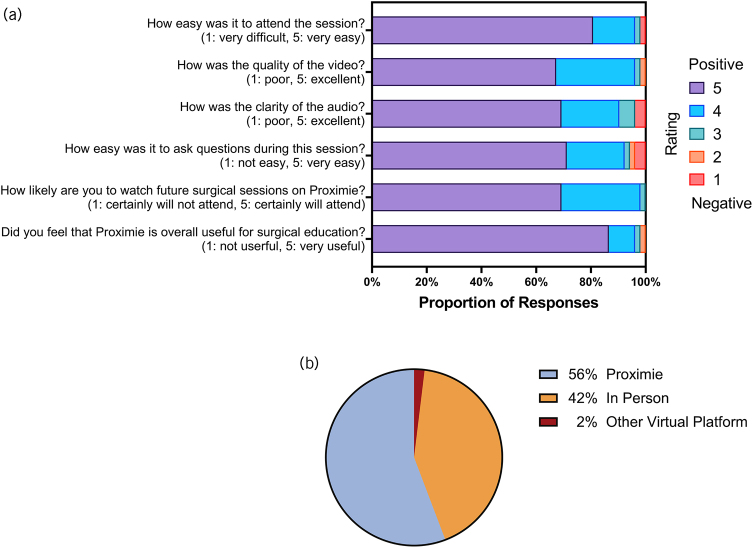
(a) Bipolar descriptor questions (on a scale of 1–5) pertaining to Proximie as a surgical education platform. Ratings of 5 are the most positive, whereas ratings of 1 are the most negative. (b) Percentage of responses that preferred Proximie, in person, or free text – of which one respondent put ‘Other Virtual Platform’ – to access surgical operations.

The feedback we received about Proximie was overwhelmingly positive. We asked several questions that pertained specifically to Proximie's viability as a surgical education tool. A summary of these results is shown in Figure 2. For the purposes of summary statistics in the following two sections, a positive rating was deemed as being a response ≥4 of 5.

**Figure 3:**
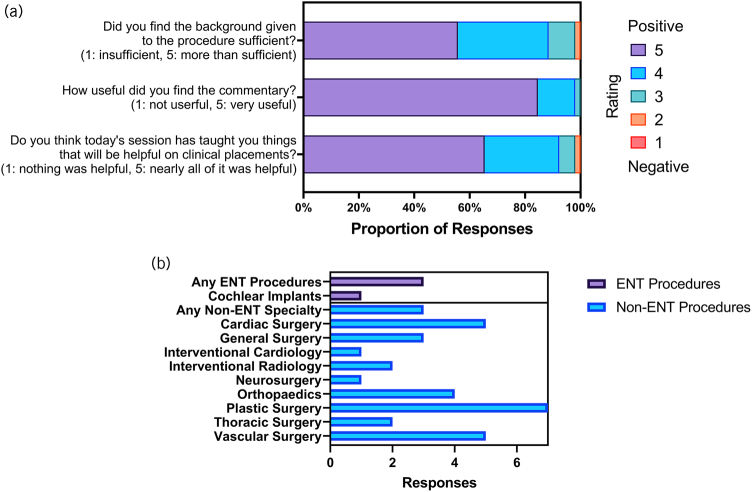
(a) Questions (on a scale of 1–5) pertaining to the quality of the ear, nose and throat (ENT) education delivered through Proximie. Ratings of 5 are the most positive, whereas ratings of 1 are the most negative. (b) Reported interest in seeing future surgical procedures sorted by ENT and non-ENT surgical procedures. Responses were free text and sorted into different categories.

Ninety per cent of all the responses to Proximie-specific questions gave a positive rating of ≥4 (Figure 2a). Nearly all (96%) of the participants found it easy to attend the session, with only two responses reporting technical difficulties. Participants had a good experience with the quality of the video and the clarity of the audio, with 90% and 96% of responses rating positively for audio and video quality, respectively. Regarding the likelihood of attending future Proximie sessions, 98% of students responded with a score ≥4 of 5. Crucially, 96% of respondents gave a positive response regarding whether Proximie was useful for surgical education and 56% of student attendees reported a preference for Proximie over attending the operating theatre in person or using other virtual operating platforms (Figure 2b).

We received six free-text responses as to why students preferred Proximie for attending the operating theatre and six responses as to why students preferred to attend in person (Appendix 3A and 3B – available online). Students who preferred Proximie reported doing so because of increased comfort and convenience, better vision of the surgical field, and the ability to tune in when on non-surgical or away clinical placements. Respondents that preferred in-person theatre sessions reported doing so because it offered the opportunity to experience the operating theatre environment and to scrub in to procedures.

### Surgical teaching and future procedures

The responses we received regarding the quality of surgical teaching were all very positive. These questions included whether the background was sufficient, whether the commentary was useful and whether the theatre session taught students helpful and relevant information. Of all the 1–5 scale questions, students gave the most positive response to the quality of the commentary, with 98% responses rating the commentary ≥4 of 5. Students also felt that teaching ENT procedures taught relevant material, with 94% of responses being positive. In addition, 88% of responses were positive with respect to background given before a surgical procedure (Figure 3a).

Students were also asked what additional ENT and non-ENT surgical procedures they would be interested in having live streamed using Proximie in the future (Figure 3b). Students reported an interest in seeing more ENT procedures, whereas responses to non-ENT procedures ranged widely from cardiac surgery to general surgery, orthopaedics and endovascular procedures. The three specialties the students reported the most interest in seeing were plastic surgery, cardiac surgery and vascular surgery.

When we asked students what they found most helpful, they most frequently cited the commentary by surgeons and communication from the session organisers. Students felt that these sessions could be improved by increasing advertising, communicating the theatre lists the night before, giving microphones to multiple surgeons, and broadening the surgical specialties that were filmed (Appendix 3C and 3D – available online).

## Discussion

Given the practical nature of surgery, virtual sessions can never fully replicate the operating theatre. Basic surgical skills, such as scrubbing in, tissue handling, suturing and knot-tying, require hands-on experience. However, the results of this study illustrate that Proximie can be used effectively alongside in-person theatre sessions to teach students about common procedures in ENT. Most notably, more students preferred Proximie to in-person teaching or other online videos. Responses to all other questions pertaining to Proximie's use in surgical education were overwhelmingly positive. Students also appeared interested in seeing surgical operations from other departments. If offered in this broad specialty-wide format, Proximie has the potential to increase access to surgery by breaking down the traditional barriers that prevent students from entering the operating theatre.

### Study limitations

There are some limitations to this work. We acknowledge that our approach relied primarily on gathering students' perceptions about the surgical experience, rather than objectively assessing their knowledge acquisition or retention. This emphasises Proximie's success at the first level of the Kirkpatrick model, which looks at reactions and attitudes towards the learning experience. Future work should include objective metrics to evaluate Proximie's impact on surgical knowledge gained and its role in achieving medical school exam outcomes in clinical skills assessments or applied knowledge examinations.

In addition, we only received 52 responses over four theatre days, despite the organisers urging students to respond after each session they attended. Many attendees did not submit a response to each session they attended. Many students who were repeat attendees did not respond to the survey more than once. Moreover, there may be considerable selection bias in the responses we received. This is because students had to first register their interest in attending ENT surgical teaching before attending. Attendance was not timetabled in the medical school curriculum and thus competed with clinical and extra-curricular commitments. Students who were already reasonably interested in surgery may have been more likely to respond positively and more frequently to the questionnaire. The relatively small data set and potential for selection bias in responses prompt us to approach generalisation cautiously.

The biggest learning points from this pilot study pertain to attendance and logistics. Attendance at these live sessions dropped off after the first session. The increased participation in the last session likely owes to the start of a new academic year and new medical students entering their first clinical year. Many operations on the second and third theatre day only had one or two students attending virtually. Attendance could be improved by providing more notice to students regarding procedures and including these sessions as a part of the medical school timetable. Proximie sessions also necessitate a coordinator: someone to help set up equipment and coordinate surgeons and attending students when filming. This scheduling can prove difficult when a medical student or clinical trainee must fit their attendance in around their other commitments. Future sessions should involve recruitment of more Proximie coordinators to allow for an increased number of sessions. Recorded sessions may be useful as an alternative or additional resource in the future as an adjunct to in-person surgical teaching. This way, specific lists with commentary by interested surgeons could allow for the best possible conditions to film a theatre list. In addition, resources could be focused on making the best possible recordings just once and allow for a growing bank of operations to be used for years to come, rather than necessitating a continuous effort on the part of surgeons and Proximie student coordinators.

## Conclusions

Proximie serves as a valuable tool in surgical education, as evidenced by student reports, by enhancing student engagement and enabling surgeons to extend their teaching beyond in-person methods. It achieves this by providing students with increased exposure to procedures across various surgical subspecialties. To maximise attendance at these sessions, medical schools should consider advertising and seamlessly integrating them into the official clinical timetable. In the future, Proximie holds the potential to create a repository of surgical procedures from diverse specialities to allow students to observe procedures they may have otherwise missed because of surgical firm allocations. Operating theatre lists could be specifically tailored to medical students and potentially for core surgical trainees as well. Future work assessing Proximie's effectiveness in teaching surgical competencies across various stages of medical student and surgical trainee progression should be evaluated. Ultimately, integrating Proximie into medical school curricula has the potential to be extremely effective and fruitful. It stands to significantly enhance the quality of surgical education and foster increased interest in surgical specialities.

## **Appendix 1** Proximie setup

**Table:**

Product Name	Use
Dell i7 Laptop	Computer
Video Option	Webcam or Pant-tilt-zoom (PTZ) – captures surgical field and/or table (specific camera to be determined by account or account manager)
C-Clamp	Used to mount cameras. One per camera included (works w/PTZ as well)
USB Extenders	Allows speaker or webcam to be placed maximum of 20 feet away from laptop
Microphone Option	Jabra Headset with Bluetooth headset
Speaker	JBL or Jabra Puck (specific speaker to be determined by account or account manager)
Capture Cards	Connects devices to the laptop via DVI, HDMI, or SDI - one capture card required per input (excl. video/audio)

## **Appendix 2:** Proximie Questionnaire

Italics indicate labels that were put in the survey to guide responses using a semantic differential scale using bipolar alternatives.

**Table:**

Question	Options
Medical Student Status	Undergraduate, Graduate-Entry
Medical Student Year	1, 2, 3, 4, 5, 6
Date of Procedure	Free Text
Consultant Name	Free Text
Did you find the background sufficient?	1, 2, 3, 4, 5 *1: Insufficient, 5: Sufficient*
How easy was it to attend the session?	1, 2, 3, 4, 5 *1: Insufficient, 5: Sufficient*
How was the quality of the video?	1, 2, 3, 4, 5 *1: Insufficient, 5: Sufficient*
How was the clarity of the audio?	1, 2, 3, 4, 5 *1: Insufficient, 5: Sufficient*
If there was an introductory video, did you find it useful?	1, 2, 3, 4, 5 *1: Insufficient, 5: Sufficient*
How useful did you find the commentary?	1, 2, 3, 4, 5 *1: Insufficient, 5: Sufficient*
What is your preferred way to watch procedures, and why?	In Person, Proximie, YouTube (other pre-recorded procedures), then Free Text
Why do you prefer to use the aforementioned resource?	Free Text
How easy was it to ask questions during the session?	1, 2, 3, 4, 5 *1: Not easy, 5: Very easy*
How likely would you watch future surgical sessions on Proximie?	1, 2, 3, 4, 5 *1: Certainly will not attend, 5: Certainly will attend*
Did you feel that Proximie is overall useful for surgical education?	1, 2, 3, 4, 5 *1: Not useful, 5: Very useful*
Are there any other procedures in ENT that you would like to see?	Free Text
Which other surgical specialties would you be interested in seeing procedures from?	Free Text
Do you think today's session has taught you things that will be helpful on clinical placements?	1, 2, 3, 4, 5 *1: Nothing was helpful, 5: Nearly all of it was helpful*
Please list what you found most helpful about this Proximie session	Free Text
What could we do to make these sessions even better?	Free Text
Do you have any other feedback for us?	Free Text

## Appendix 3: Proximie Comment Question Responses

Appendix 3A

**Table:**

**Why do you prefer to use Proximie over other surgical teaching resources?**
Ease and convenience.
It is more convenient than turning up in person and waiting around for the surgery to start. I'd prefer online for endoscopic/laparoscopic procedures, but in person for open procedures.
It's easier to see – often for close-up procedures when watching in the room as a medical student you can see very little, whereas this way the anatomy is much easier to see.
It's easier to see what's happening and hear about what they are doing.
It's more comfortable than standing/sitting in theatre, and you get a better view of the cameras in my opinion.
Proximie so ideal when in person isn't possible for whatever reason (e.g. time/ capacity). Also means that if not on a surgery attachment, can still access ENT content. Arguably, Proximie gives better visualisation of endoscopic procedures than in real life as you can essentially get closer to the screen.

## Appendix 3B

**Table:**

**Why do you prefer to be in surgical theatre rather than virtual theatre settings?**
In person is optimal to appreciate the start to finish and the nuances to surgery. However, in order to maximise procedures seen, Proximie is better as you can dip in and out throughout the day.
Occasionally I am offered the opportunity to scrub into the procedure.
Nice to see the atmosphere.
Get the holistic ‘vibe’ of theatre, can assist, *sometimes* better vision (though sometimes not- which is where Proximie could be really good!!).
Environment is nicer in person.

## **Appendix 3C:** Names of surgeons are redacted.

**Table:**

**Please list what you found most helpful about this Proximie session.**
Constant commentary from surgeon.
Communication in the WhatsApp chat was very clear, lots of notice given, very easy to access.
Good view of procedure and running commentary from surgeon.
Was really easy to visualise and learn ENT anatomy and the flexibility in attending and only needing to be online whilst the procedure was happening, rather than a whole day's list was really great.
Very nice explanations of the different ways that the ear drum can be repaired and also gave a good overview of the procedure itself.
Very helpful commentary. Good vision.
Nice to see ENT procedures which are different from other procedures we usually get (eg appendectomy).
Seeing the cholesteatoma macroscopically.
Good commentary from the registrar and questions from the others in the room. (medical student?)
Good commentary and view of the field.
Interactivity and ability to ask live questions.
Nice to see exactly what the grommets look like and to see how the pus from glue ear can be removed.
Surgeons discussing through their thought processes, reasoning for techniques, planning equipment, and recapping anatomy and pathology knowledge.

## Appendix 3D

**Table:**

**What could we do to make these sessions even better?**
Advertise further within the medical school – I feel that a lot more students would be interested in participating if given repeated notice that the day is virtual and easily accessible at any point during their day.
Do more of them with more specialties.
Maybe send the case list out the night before – I would have really liked to see some of the surgeries that were on earlier in the day and had I known I could have maybe slightly changed my schedule for the day.
Could put microphones on multiple people so we can hear the consultant and the registrar discuss.
Had some problems at the start with the audio cutting off.
Some more introductory background would be useful.
The consultant asked us questions at the end of the operation. Unfortunately, I couldn't respond for some reason. It could have been a tech issue from my end, but no one else responded either, which made me think it was an issue from your end?
Was mostly for interest, I didn't learn a lot. But perhaps this is because my audio wasn't working so I couldn't hear the commentary.
